# Working conditions in low risk nulliparous women in The Netherlands: are legislation and guidelines a guarantee for a healthy working environment? A cohort study

**DOI:** 10.1007/s00420-022-01888-y

**Published:** 2022-06-16

**Authors:** Monique D. M. van Beukering, Heleen J. Schuster, Myrthe J. C. S. Peelen, Marit E. A. Schonewille, Petra J. Hajenius, Ruben G. Duijnhoven, Teus Brand, Rebecca C. Painter, Marjolein Kok

**Affiliations:** 1 Department of Obstetrics and Gynaecology, Location Amsterdam Medical Center, Amsterdam University Medical Centres, P.O. Box 22660, Amsterdam, 1100 DD The Netherlands; 2 Department of Medical Microbiology and Infection Control, Amsterdam UMC, Vrije Universiteit Amsterdam, Amsterdam, The Netherlands; 3 Coronel Institute of Occupational Health, Amsterdam Public Health Research Institute, Amsterdam, Amsterdam UMC, University of Amsterdam, Amsterdam, The Netherlands

**Keywords:** Employment, Maternity protection, Occupational exposure, Pregnancy, Protective legislation, Preterm birth

## Abstract

**Objective:**

Hazardous working conditions increase the risk of adverse pregnancy outcomes. In this study, we examine adherence to legislation and guidelines aimed at improving working conditions in pregnancy.

**Methods:**

Between 2014 and 2016, we recruited a prospective cohort of low-risk nulliparous pregnant women in paid employment or self-employed in 16 community midwifery practices in The Netherlands. Participants completed two questionnaires concerning demographics, education, general health and working conditions between 10–16 and 20–24 weeks of pregnancy. We calculated the proportion of participants with work-related risk factors not in accordance with legislation and/or guidelines.

**Results:**

Of 269 participants included, 214 (80%) completed both questionnaires. At 10–16 weeks 110 (41%) participants and at 20–24 weeks 129 (63%) participants continued to work under circumstances that did not meet recommendations. Employers provided mandated information on work adjustment to 37 (15%) participants and 96 (38%) participants received no information about the potential hazards while working with biological and chemical hazards. Participants with lower educational attainment (aOR 2.2 95%CI 1.3–3.9), or employment in healthcare (aOR 4.5, 95%CI 2.2–9.0), education/childcare and social service (aOR 2.6, 95%CI 1.1–6.0 2),, catering (aOR 3.6, 95%CI 1.1–12) and industry, construction and cleaning (aOR 3.3, 95%CI 1.1–10.3) more often continued work which did not meet recommendations.

**Conclusion:**

There is poor adherence to national legislation and guidelines for safe working in pregnancy in The Netherlands: 50% of the pregnant women worked under hazardous conditions. Given the impact on adverse pregnancy outcomes as well as on the public purse, action to improve compliance must be taken by all stakeholders.

## Introduction

Many women continue their paid job during pregnancy. In the US, 66% of mothers who gave birth to their first child between 2006 and 2008 worked during their pregnancy (Laughlin 2011). Hazardous working conditions, including physically demanding work, long working hours and high job strain, may increase the risk of adverse pregnancy outcomes such as miscarriage (Cai et al. 2020, 2019; Croteau 2020; Bonde et al. 2013 Jul), hypertensive disorders (Cai et al. 2020, 2019; Brandt and Nielsen 1992; Zhang, et al. 2013), foetal growth restriction (Cai et al. 2020, 2019; Brandt and Nielsen 1992; Lee et al. 2011; Oths et al. 2001; Vrijkotte et al. 2009; Selander et al. 2019), preterm birth (PTB) (Cai et al. 2020, 2019; Croteau 2020; Beukering et al. 2014; Melick et al. 2014; Vrijkotte et al. 2021) and foetal abnormalities (Snijder et al. 2012; Zheng et al. 2015; CDC 2022; Allotey et al. 2019) (Supplement A). Two systematic reviews, including 80 observational studies on work-related adverse pregnancy outcomes, show that various types of physically demanding work, shift work and working > 40 h per week increased the odds of PTB by 10 to 31%. Furthermore, lifting > 10 kg, fixed night shifts and working > 40 h per week increased the odds of miscarriage by 35%, 23% and 38%, respectively (Cai et al. 2020, 2019). These adverse pregnancy outcomes can be prevented by work adjustment. Elimination of harmful work-related exposures before 24 weeks of pregnancy through implementation of legal measures was shown to result in a 30 to 50% reduction in risks for PTB (Croteau et al. 2007) and foetal growth restriction (Croteau et al. 2006).

Maternity protection legislation (MPL) and evidence-based guidelines on working conditions in pregnancy are available in many countries (Probst et al. 2018; International Labour Organization, 2010). Recommendation in MPL include restricted work time (night work and overtime) and provisions on hazardous work, and are generally aimed at prevention of adverse pregnancy outcomes. Common principles pertaining to the topic of work and pregnancy were recorded by The International Labour Organisation (ILO) with information derived from 111 countries. These principles include: (1) risk assessment and providing pregnant employees with information about these risks; (2) workplace adjustments or temporary assignment of pregnant employees to a job without risk for pregnancy complications; (3) temporary leave, preferably with retention of financial compensation for the employee (International Labour Organization, 2010, 2014). The implementation of MPL is lacking in most countries (Probst et al. 2018) and pregnant women continue to work in a hazardous workplace or resort to sick leave.

In The Netherlands, 9 in 10 women are in paid employment and continue to work in their first pregnancy (Portegijs and (SCP)^2018^). Legislation and guidelines are available to ensure a safe workplace for pregnant women. European Union law requires employers to perform an occupational risk assessment regarding pregnancy, according to Council Directive 92/85 / EEC (Agency and for Safety and Health at work, 2022). Employers are required to provide their employees, who wish to become or are pregnant, with information on work adjustment and enable them to continue work in a safe environment. In addition to European legislation, occupational physicians from The Netherlands Society of Occupational Medicine (NVAB) in collaboration with other experts in the field have developed an evidence-based guideline ‘*Pregnancy, Postpartum Period and Work’*. This guideline includes recommendations regarding various work-related risk factors enabling occupational physicians to advise pregnant employees (with or without pre-existing health problems or pregnancy complications) and their employers on work adjustment (NVAB 2018). Finally, the Dutch Social and Economic Council (SER) has drawn up a ‘*Guide to Occupational Health and Safety Measures Pregnancy & Work*’ for employers and employees to make the workplace safer and healthier for pregnant women within individual organizations (Social and Economic Council of the Netherlands (SER). 2018). To date, the implementation of legislation and guidance on working conditions and the effect on pregnancy is unknown.

The aim of this study was to examine whether the Dutch MPL and guidelines have been implemented and if not, which work-related risk factors are involved in adverse pregnancy outcomes.

## Methods

### Design

We used data from the PROPELLOR (PRevention Of PrEterm Labor in LOw Risk women) study, a cohort study in a population of low-risk nulliparous women to identify risk factors associated with spontaneous PTB between 16 and 37 weeks of pregnancy (Schuster et al. 2022). Pregnant women were recruited at 16 midwifery practices in the region North-West Netherlands between February 2014 and December 2016. The study was approved by the Medical Ethics Committee of the Amsterdam University Medical Centre, location Amsterdam Medical Centre (registration number NL43414.018.13).

### Legislation and guidelines

We used the guideline *‘Pregnancy, Postpartum Period and Work’* (NVAB 2018) and the ‘*Guide to Occupational Health and Safety Measures Pregnancy & Work*’ (Social and Economic Council of the Netherlands 2018) both of which include legislation. We distinguished work-related risk factors for adverse pregnancy outcomes before 20 weeks of pregnancy and from 20 weeks onwards; we defined these work-related risk factors as > 40 h/week,  ≥ 4–6 h/day standing and walking, lifting > 5 kg > 10–50 times/day, very physically demanding regularly/ often, bending regularly/often, squatting regularly/often, high work pressure regularly/often, working in noise and work at night. The exact limits of these risk factors before and after 20 weeks pregnancy are listed in Table 1.

**Table 1:** Risk factors in work that exceed the limit values of guidelines and legislation (from The Netherlands Society of Occupational Medicine and Social Economic Council)

< 20 week pregnancy	≥ 20 weeks pregnancy
> 40 h/week ≥ 6 h/day standing + walking/day + rarely/never possible to sit Lifting > 10 kg > 50 times/day Very physically demanding: often Bending down: often Squatting: often Problems with the pressure: regularly/ often Working in noise: often	> 40 h/week ≥ 4 h/day standing + walking Lifting > 5 kg > 10 times/day Very physically demanding: regularly/ often Bending down: regularly/often Squatting: regularly/often Problems with the pressure: regularly/ often Working in noise: often Work at night

We constructed a cumulative work risk variable, with which we compared ‘working in accordance with legislation and guidelines’ (score = 0 risk factors) with ‘working in the presence of ≥ 1 risk factors’ (score = 1–8 at 10–16 weeks of pregnancy and score = 1–9 at 20–24 weeks of pregnancy).

### Participants

The PROPELLOR study included nulliparous adult women with a low-risk pregnancy, being healthy women with no co-morbidity at antenatal booking between 8 and 12 weeks of pregnancy. Women were followed-up until delivery. For the present study, only participants with paid employment or self-employment, and who had completed at least the first of two questionnaires were eligible. All participants provided written informed consent.

### Data collection

All participants were asked to complete two questionnaires: a questionnaire between 10 and 16 weeks and a questionnaire between 20 and 24 weeks of pregnancy. Questionnaires were either completed on paper or online via a website developed for the PROPELLOR study. All data were collected on web based electronic case report forms, and were stored in an anonymised database.

The first-trimester questionnaire between 10 and 16 weeks of pregnancy collected data including demographics, education, general health, lifestyle and current pregnancy. In addition, we used questions from a validated questionnaire about psychosocial job strain and physically demanding work (Vrijkotte et al. 2009) supplemented with questions about other working conditions (e.g. (irregular) working times, chemical, biological and physical factors (noise, climate)). Information on biological agents was retrieved from questions about working with ill/small children, sick adults, blood and other bodily fluids and/or stools. Furthermore, we asked whether the participant came into contact with chemical substances: cleaning supplies, solvents, anaesthetic gases, cancer-inhibitory medication, pesticides and/or heavy metals. Finally, we asked whether the employer had provided advice on how to adjust her work while pregnant. To determine the influence of private factors on health and work capacity, the last part of the questionnaire concerned commuting, sports, hobbies, and household characteristics.

The second trimester questionnaire between 20 and 24 weeks of pregnancy was used to collect work status and adjustment, working conditions, recommendations regarding work and physical and/or obstetrical complaints.

We collected participants’ antenatal files retrospectively via the midwifery and hospital practices. Medical records were used to collect data on miscarriage and/or termination of pregnancy and medical history. The socio-economic status (SES), was estimated on the postal code of residence and the status scores from The Netherlands Institute for Social Research.

### Outcome measurements

The primary outcome was the proportion of pregnant women exposed to work-related risk factors that exceed the limit values of legislation and guidelines. We distinguished between the periods before and after 20 weeks of pregnancy. Secondary outcome was the proportion of pregnant women with work-related exposure to biological and chemical agents without advice from the employer concerning safety measures.

### Statistical analysis

Baseline characteristics are presented as absolute numbers and percentages for categorical variables and means with standard deviation or median with range for continuous variables. To address the potential non-response bias, we compared baseline characteristics of responders to those of non-responders.

Work-related risk factors, as defined in Table 1, were participant-reported and retrieved from the questionnaires (supplement B). These categorical and numerical variables were converted into binary variables. The risk factor ‘standing and walking’ was constructed from two questions (hours standing and walking per day and possibility to sit), while other risk factors were based on one question each. We constructed a cumulative work risk variable, which scored a point for each work-related risk factor present (Table 1), and otherwise was scored zero if working conditions were all in accordance with the guidelines. The cumulative work risk variable was dichotomised, comparing no risk factors present (cumulative work risk variable = 0) to ≥ 1 risk factors present (cumulative work risk variable 1–8 at 10–16 weeks of pregnancy and 0 versus 1–9 at 20–24 weeks of pregnancy).

The missing values of the risk factors were imputed based on job, employment sector and the answer to the question “possibility to sit”. In the missing values of the second trimester questionnaire, the answers from the first-trimester questionnaire were included, if available. Missing data on one or more of the work-related factors were imputed in 13 (first-trimester questionnaire) and 18 participants (second trimester questionnaire). After imputation, in both questionnaires, five work-related risk factors remained missing in two and three participants, respectively. Since these participants all had a cumulative risk score of ≥ 1 risk factors, without the missing data, they were included in the analyses.

We determined the association between the cumulative work risk score and the variables educational level, number of employees in the company and employment sector, by calculating the crude odds ratio (OR) and 95% confidence intervals (CI). P-values were calculated using a chi-squared test. A *p*-value < 0.05 was considered statistically significant. Using logistic regression ORs were adjusted for SES (low or middle/high), education (primary or secondary school, lower professional versus university or higher vocational education), age (< 30 versus ≥ 30), and ethnicity (non-white European versus white European). These variables were chosen as representative for several risk factors associated with sociodemographic features. The employment sector with the lowest number of risk factors, government, business services and culture & recreation’, was chosen as reference.

Data were analysed using IBM SPSS Statistics 25 (Statistical Package for the Social Sciences, SPSS Inc., Chicago, IL, USA) for Windows.

## Results

**Fig. 1:**
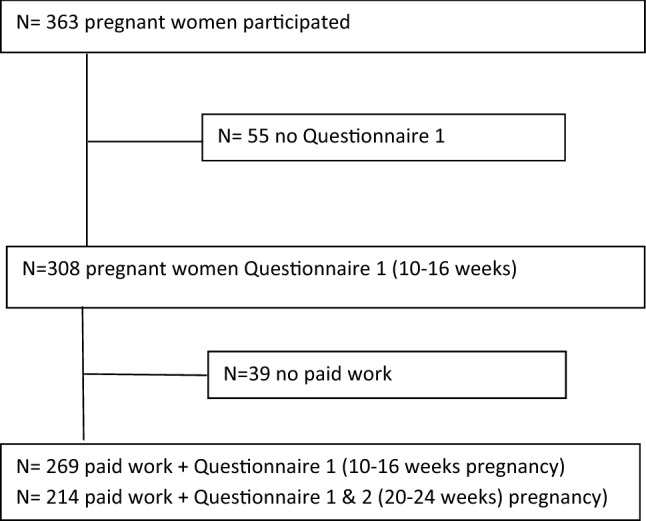
Flow chart PROPELLOR Study

A total of 363 participants were enrolled in the PROPELLOR study, the first-trimester questionnaire was completed by 308 participants, of whom 39 without paid work. In this study, we included a total of 269 women with paid employment or self-employed, of whom 214 (80%) completed both questionnaires (Fig. 1).

Mean maternal age was 29 (SD 4.2) years, the median body mass index (BMI) was 23.7 (SD 4.1) kg/m^2^, 208 (77%) women were white European, 82 (31.3%) had a low SES, 173 women (64%) had completed tertiary or higher vocational education. Ten (4%) women were single, six (2%) cared for their own or other children and nine (3%) had a previous medical disease (Table 2).

**Table 2:** Baseline characteristics study population: of nulliparous working women with a low-risk pregnancy at 10–16 weeks

Demographics and general health	*N* = 269	Work: General aspects	
Age (years)	29.1 (4.2)*	Paid work from start of the pregnancy	264 (98.1%)
Age < 30 (versus ≥ 30)	138 (51%)	Paid work from x weeks pregnancy	5 (1.9%) x = 8.8 (4–13%)
Body Mass Index (kg/m2)	23.7 (4.1)*	Working in sector	
Ethnic origin: White European	208 (77%)	Health care	78 (29%)
University or higher vocational education	173 (64.3%)	Business services	66 (24.5%)
Low SES score	82 (31.3%)^a^	Education	25 (9.3%)
Smoking during pregnancy	12 (4.5%)	Retail	22 (8.2%)
Someone smoked at work last 30 days	36 (13.4%)	Culture, recreation	16 (5.9%)
Alcohol during pregnancy	2 (0.7%)	Government	16 (5.9%)
Drugs during pregnancy	5 (1.9%)	Social service and child care	15 (5.6%)
Physical activity during pregnancy (sports)	118 (43.9%)	Hospitality and Catering	15 (5.6%)
Medical history: No previous disease	253 (96.6%)^b^	Industry/ construction	10 (3.7%)
Medication prescribed by physician	38 (14.1%)	Cleaning	5 (1.9%)
Health complaints before pregnancy limit work	20 (7.4%)	Number of employees in the company	
Pregnancy characteristics		1–10	48 (17.9%)
Previous pregnancy:		11–50	66 (24.5%)
1–10 miscarriage and/or abortion: 55/262	55 (21%)^b^	51–100	19 (7.1%)
11–50 Increase in complaints from current pregnancy that limit work	108 (40%)	> 100	135 (50.4%)
51–100 Fatigue	89 (33%)	Self-employed	14 (5.2%)
Self-employed Nausea/vomiting	54 (20%)	Commuting	
Headaches	30 (11%)	Travel distance commuting (m/km) *	35 (37)
Stomach aches	24 (8.9%)	Travel time commuting (min/hours) *	57 (41)
Private conditions		Travel time commuting (min/hours) **	0–300
Marital status: Single	10 (3.7%)	Means of travelling/ transport	
Care for other children living at home	6 (2.2%)	Car	140 (52%)
Housekeeping: Largely doing by participant herself	70 (26%)	Public transport	68 (25.4%)
Household help	42 (15.6%)	By bicycle/scooter	44 (16.4%)
		Walking	15 (5.6%)

*Mean (SD), ** min–max, all other variables mentioned as *N* (%)

^a^8 missing, b: 7 missing

Almost one third (*n* = 78, 29%) worked in healthcare, one quarter (*n* = 66, 25%) in business services, nearly 10% (*n* = 25) in education and 8% (*n* = 22) in retail. The average travel time commuting was 57 (± 41) minutes, 140 (52%) travelled by the car. Before pregnancy, 20 (7%) women had adjusted their work because of health problems or illnesses.

Of the 55 participants who did not complete Questionnaire 1, data retrieved from the participants’ antenatal files demonstrated that 9 had no paid work (Supplement C). The other 46 participants who did have paid work (but did not complete Questionnaire 1), were comparable to the study population in age (29) and BMI (23.6 vs. 23.7), the number with a Low SES score was lower in the non-response group (20% vs. 31%) (Supplement C).

Before 20 weeks of pregnancy, 110 (41%) women continued to work under circumstances that were not in accordance with the Dutch guidelines and legislation. From 20 weeks of pregnancy, this number was 129 (63%) (Table 3).

**Table 3:** Number of work-related risk factors in the work of pregnant women that exceeds the limit values of guidelines and legislation (from The Netherlands Society of Occupational Medicine and Social Economic Council)

Risk factors*	< 20 weeks pregnancy *N* = 269	≥ 20 weeks pregnancy *N* = 214, 205 at work
No	159 (59%)	76 (37%)
Yes	110 (41%)^**a**^	129 (63%)^b^
1	56 (21%)	44 (22%)
2	27 (10%)	24 (12%)
3	13 (5%)	23 (11%)
4	10 (4%)	15 (7%)
5	3 (1%)	15 (7%)
6	1 (0.4%)	6 (3%)
7		2 (1%)

^*^All variables mentioned as N (%)

^a^Missing *n* = 5 work-related risk factors of *n* = 2 participants, both with ≥ 1 risk factors

^b^Missing *n* = 5 work-related risk factors of *n* = 3 participants, all with ≥ 1 risk factors

Specification of the risk factors that exceeded the limit values of guidelines and legislation is shown in Table 4. Frequent bending down (*n* = 44, 17%) and problems with job strain (*n* = 43, 16%) were the most frequently exceeded risk factors before 20 weeks of pregnancy. From 20 weeks of pregnancy standing and/or walking ≥ 4 h a day was the most frequently observed risk factor in excess of guideline, occurring in 88 (43%) women followed by bending regularly in 65 (32%) and very physically demanding work in 47 (23%).

**Table 4:** Specification of risk factors in the work of pregnant women, that exceeds the limit values of guidelines and legislation (from Netherlands Society of Occupational Medicine and Social Economic Council)

< 20 weeks pregnancy *N* = 269, all at work ^a^		≥ 20 weeks pregnancy *N* = 214, 205 at work ^b^	
> 40 h/week	19 (7%)	> 40 h/week	16 (8%)
Very physically demanding: often	32 (12%)	Very physically demanding: regularly/ often	47 (23%)
≥ 6 h standing + walking/day and rarely/never possible to sit	25 (8%)	≥ 4 h standing + walking/day	88 (43%)
Bending down often	44 (17%)	Bending down regularly/often	65 (32%)
Squatting often	32 (12%)	Squatting regularly/often	45 (22%)
Lifting > 10 kg > 50 times/day	6 (2%)	Lifting > 5 kg > 10 times/day	41 (20%)
Problems with job strain: regularly/ often	43 (16%)	Problems with job strain: regularly/ often	33 (16%)
Working in noise: often	14 (5%)	Working in noise: often	8 (4%)
		Work at night	7 (3%)

*All variables mentioned as *N* (%)

^a^Missing *n* = 5 work-related risk factors

^b^Missing *n* = 5 work-related risk factor

Exposure to biological or chemical agents occurred in 127 out of 269 (47%) women of whom 117 were in employment and 10 were self-employed (Table 5). Of women in employment, 37 (15%) were informed about work adjustments by their employers and 22 (8%) by their obstetric healthcare provider. There was lack of information about biological and chemical exposure provided by the employer in 96 (38%) cases.

**Table 5:** Biological and chemical exposure and advice to adjust work, from pregnant workers < 20 weeks pregnancy and/or ≥ 20 weeks pregnancy, *n*= 269

Biological and chemical exposure*	*N* = 269
Exposure	127/269 (47.2%)
Exposure + in employment	117/255 (46%)
Exposure + self-employed	10/14 (71%)
Advice to adjust work*	
Advice to adjust work from:	
Employer/ supervisor^a^	37/255 (14.5%)
Midwife/ obstetrician	22/269 (8.2%)
Biological and chemical exposure + advice from management^a^	21/255 (8.2%)
Biological and chemical exposure without advice from management^a^	96/255 (37.6%)

*All variables mentioned as *N* (%)

^a^Participants in employment, self-employed women excluded

The association between the cumulative work risk score and the variables SES, educational level, age, ethnicity, number of employees in the company, employment and sector is shown in Table 6; effect estimates have been adjusted for SES, educational level, age, and ethnicity. In the first trimester, participants with lower educational level more often had a cumulative work risk score from 1 to 8, than those with higher educational level (aOR 2.2 95%CI 1.3–3.9), meaning they more frequently continued to work under circumstances that were not in accordance with the Dutch legislation and guidelines. Also participants with an age < 30 (versus ≥ 30 years) more often had a cumulative work risk score 1–8 (OR 1.9, 1.2–3.2), after adjustment this association was not significant. Neither SES, ethnicity, the number of employees in a company nor being self-employed impacted the cumulative work risk score.

**Table 6:** Logistic regression Cumulative work risk score 1–8 vs 0, in the work of pregnant women with educational level, number of employees in the company, employment and sector^a^

Participants: 10–16 weeks pregnancy *N* = 269, all at work	Cum. work risk score: 1–8	OR	(95% CI)		*P* value	aOR	(95%CI)	*P* value
SES								
Low	32 (39%)	0.87	0.51–1.48		0.601			
Middle and High (ref.)	76 (43%)							
Education								
Primary or secondary school, lower professional	54 (56%)	2.67	1.61–4.49		0.000	2.2	1.26–3.91	0.006
Higher vocational or University (ref.)	56 (32%)							
Age								
< 30	67 (49%)	1.93	1.18–3.17		0.009	1.5	0.85–2.65	0.157
≥ 30 (ref.)	43 (33%)							
Ethnicity		1.36	0.76–2.44		0.301			
Non-white European	28 (48%)							
White European (ref.)	81 (40%)							
Number of employees in the company								
1–50 employees	53 (47%)	1.52	0.93–2.49		0.096			
> 50 employees (ref.)	56 (36%)							
Employment								
Self-employment	6 (43%)	1.09	0.37–3.32		0.88			
In company (ref.)	104 (57%)							
Sector								
Government, business services, culture & recreation	22 (22%)	Ref				Ref		
Health	44 56%)	4.47	2.33–8.59		0.000	4.53	2.28–9.0	0.000
Education, child care and social service	16 (40%)	2.3	1.04–5.08		0.039	2.62	1.14–6.04	0.024
Retail	11 (50%)	3.46	1.32–9.03		0.011	2.25	0.82–6.149	0.226
Hospitality and Catering	9 (60%)	5.18	1.66–16.15		0.005	3.63	1.1–11.97	0.034
Other, Industry, construction, cleaning	8 (50%)	3.46	1.16–10.26		0.026	3.34	1.08–10.34	0.036

*All variables mentioned as *N* (%)

^a^Cumulative work risk score: 1–8 risk factors versus 0 risk factors

*aOR* odds ratio adjusted for socio-economic status, education, age and ethnicity

*Ref*. Reference group

A cumulative work risk score of 1–8 was more often present in women working in healthcare (OR 4.5, 95%CI 2.3–8.6), education, childcare and social service (OR 2.3, 95%CI 1.04–5.1) retail (OR 3.5, 95%CI 1.3–9.0), hospitality and catering (OR 5.2, 95% CI 1.7–16.2), and industry, construction and cleaning (OR 3.5 95% CI 1.2–10.3) compared to the reference employment sector 'government, business services and culture & recreation’ (Table 6). Adjusting did not substantially change these associations for the sector healthcare (aOR 4.5, 95%CI 2.2–9.0), education, childcare and social service (aOR 2.6, 95%CI 1.1–6.0), hospitality and catering (aOR 3.6, 95%CI 1.1–12), and industry, construction and cleaning (aOR 3.3, 95%CI 1.1–10.3). After adjustment, the association between the cumulative work risk score for the sector retail was no longer statistically significant.

## Discussion

In this study we found that between 41 and 63% of pregnant women continued to work under conditions that were not in accordance with the Dutch legislation and guidelines. In addition, 38% of women worked in an environment with infectious diseases or chemical exposure without receiving advice from the employer on safe working conditions. Only 15% of employers fulfilled their legal obligation to correctly inform their pregnant employees about work adjustments. Women with lower educational attainment, or those who worked in healthcare, education, childcare and social service, catering and industry, construction and cleaning sectors were at particular risk of continuing work in accordance with Dutch legislation and guidelines.

The strength of our study is the representative sample: we recruited a multi-ethnic sample of healthy nulliparous pregnant women with a wide range of education and SES backgrounds. Their employment was in a variety of sectors, with a wide range of working conditions. Professions and sectors in which participants were employed were reflective of national Dutch figures (CBS 2022). Although the sample size with 269 participants is limited, the response rate is high with 80% of recruited women completing both questionnaires. As the results of the baseline characters of non-responders were comparable to those of the participants of our study, we do not expect this to affect the results of the study.

A limitation of our study is that self-employed pregnant women were underrepresented (5.2%). In The Netherlands, there are no extra legal or financial provisions for these women except for a limited maternity leave benefit, which makes them even more vulnerable to compliance with MPL. Another limitation is that women completed the first questionnaire between 10 and 16 weeks of pregnancy. It is possible that employers were not yet informed of the pregnancy of their employee and therefore had not given any information about work adjustment. However, the fact that adherence to guidelines was even lower in the second trimester compared to the first trimester points to a more systematic lack of implementation of MPL. Moreover, risk of exposure to chemical, biological or radioactive agents obliges the employer to provide information about necessary measures to any of his/her employees of childbearing age who may be considering pregnancy, upon entering employment. The fact that this has not been discussed by the time of the established pregnancy indicates that employers have not adhered to this recommendations at a much earlier point in time.

Despite being prospectively collected, the data on exposure to risk factors at work are self-reported by the working pregnant women. It is therefore not entirely certain whether this reflects the actual exposure. In this study, we focussed on the implementation of Dutch guidelines for working pregnant women and did not describe whether insufficient adherence to guidelines on the topic of work and pregnancy led to an actual increase in adverse outcomes including preterm birth of foetal growth restriction. Only for a limited number of individual risk factors a reliable value of the association with some adverse outcomes has been given (Cai et al. 2020, 2019; Croteau 2020; Vrijkotte et al 2009; Beukering et al. 2014; Vrijkotte et al. 2021). The risk probably also differs per trimester (Beukering et al. 2014) and many women also have to deal with multiple exposure (walking and lifting, work stress and night shifts). It is not known whether the magnitude of the risk is the sum of the individual risk factors.

Our findings are similar to what is reported in other European studies. In a British study, 19% of 3254 mothers said that they had identified health hazards while their employer did not (Adams et al. 2016). In a Swiss online survey, comprising 2809 women who gave birth, 53% reported adjustments or change of their work but 20% did not, and only 6% received preventive leave (Rudin et al. 2018). Surveys in Poland and Norway show that 60% and 30% of working pregnant women, respectively, felt that they had not received the right job adjustment (Makowiec-Dabrowska et al. 2003; Kristensen et al. 2008). Concerning risk analysis, in a report of the British government nearly all employers (98%) claimed they undertook health and safety risk assessments for all workers and specific for pregnant women, whereas 49% of women said they were informed by their employer about risks for themselves or their baby (Adams, et al. 2016). Another Swiss study, comprising 2809 postpartum women, reports that only 26% of women felt that their employer had fully informed them about the risks in their work (Rudin et al. 2018). Implementation of legislation and guidelines appears suboptimal in several European countries, but implementation in The Netherlands displays a number of additional shortcomings in comparisons to other European countries. The prevailing standard in The Netherlands is that women are primarily responsible for caring for children, and that men are responsible for income (Portegijs and (SCP) 2018; Rabaté and Rellstab 2021). Pregnancy and maternity leave is just 16 weeks in total (20 weeks for multiple pregnancy) and a large proportion of (up to 75%) women work part-time. Mothers’ earnings are 46% lower compared to their pre-birth earnings trajectory, whereas fathers’ earnings are unaffected by childbirth. This gender stereotyping and gender norms may hamper implementation of MPL, it is not taken for granted and stakeholders are unaware of the importance.

Our study shows there is poor adherence to legislation and guidelines for safe working in pregnancy in The Netherlands. Creating greater awareness by identifying women 'at risk' and adjusting their work can prevent health care costs due to complications including PTB. This saves expenses for health insurers and benefits the society as a whole. However, companies and organisations responsible for risk analysis and work adjustment do not benefit from this 'profit'. The same applies to the costs of absenteeism due to pregnancy and childbirth, which are reimbursed to employers in The Netherlands by the Employee Insurance Agency (UWV). Reduction of these costs benefits the UWV. The lack of financial incentives for employers appears to be an important barrier to implementation as well (Sejbaek et al. 2019).

The SER identified similar bottlenecks in work-related care in general: insufficient access to occupational healthcare and attention to prevention and insufficient cooperation with regular care. Reason to start the project “Arbozorg Nieuwe Stijl” (Health and Safety New Style), an innovative form of financing and implementation of work-related care in the installation technology sector (TNO 2022). This project led to new (financing) agreements between the various stake holders and the introduction of a preventive consultation for employees. This project offers opportunities for innovative work-related care for pregnant women, in which health insurers, (regular) obstetrical and occupational health care can work together. Health insurers and society in particular benefit from the implementation of MPL, which is why they should bear the costs and responsibility for implementation together with employers. They could start organising and reimbursing the preventive consultation for working pregnant women, together with representatives from obstetrical and occupational care. An international approach within the EU (or ILO), as part of work-life balance policy, will ensure that sufficient progress is achieved in all Member States (Council of EU 2019).

The occupational physician can act as an interface between employers, health care professionals and pregnant women to improve the coordination of preventive counselling for pregnant women about their work. In 2007, the NVAB introduced the 'preventive consultation' with the occupational physician for all workers before or during the first trimester of pregnancy in the Guideline 'Pregnancy, postpartum period and work (NVAB 2018). Revision of this guideline in 2018 showed that the preventive consultation has added value, but implementation from the preventive consultation is lagging behind, which, like financing, depends on employers (Beukering and Verbeek 2010).

Future research is needed into innovative forms of financing and work-related care for pregnant working women in collaboration with prenatal care. A preventive consultation for all working pregnant women should be the start. The mHealth application 'Pregnancy and work' can serve as a tool especially for pregnant women at high risk for adverse pregnancy outcomes (Beukering et al. 2019).

## Conclusion

We found that among healthy low-risk pregnant nulliparous women in The Netherlands 50% worked under hazardous conditions, putting them at increased risk of adverse pregnancy outcomes. Only 15% of the employers provided information to their pregnant employees, despite being legally obliged to do so. The legislation and guidelines are adequate, drawn up jointly by all stakeholders, but are not enforced. Given the great impact on pregnancy outcomes as well as on the public purse, action must be taken by all stakeholders to improve compliance. Health insurers and society, in addition to employers, should also bear the costs and responsibility for the implementation of legislation and guidelines for safe working in pregnancy. The joint organisation and reimbursement of a preventive consultation for all working pregnant women, together with obstetrical and occupational care could be a practical and effective way to get started.

## Data Availability

The data are available on request.

## Abbreviations

PTB: Preterm birth
MPL: Maternity protection legislation
ILO: International labour organisation
NVAB: The Netherlands society of occupational medicine
SER: Social economic council
SES: Socioeconomic status
OR: Odds ratio
CI: Confidence interval
UWV: Employee insurance agency

## Declarations

### Disclaimer

ZonMw had no role in the study design, collection, analysis, interpretation of data, writing of the report and decision to submit the article for publication.
